# The Content and Bioavailability of Mineral Nutrients of Selected Wild and Traditional Edible Plants as Affected by Household Preparation Methods Practiced by Local Community in Benishangul Gumuz Regional State, Ethiopia

**DOI:** 10.1155/2016/7615853

**Published:** 2016-02-11

**Authors:** Andinet Abera Hailu, Getachew Addis

**Affiliations:** Ethiopian Public Health Institute, P.O. Box 5654, Addis Ababa, Ethiopia

## Abstract

Edible parts of some wild and traditional vegetables used by the Gumuz community, namely,* Portulaca quadrifida*,* Dioscorea abyssinica*,* Abelmoschus esculentus*, and* Oxytenanthera abyssinica*, were evaluated for their minerals composition and bioavailability. Mineral elements, namely, Ca, Fe, Zn, and Cu, were analyzed using Shimadzu atomic absorption spectrophotometer. Effects of household processing practices on the levels of mineral elements were evaluated and the bioavailability was predicted using antinutrient-mineral molar ratios. Fe, Zn, Ca, Cu, P, Na, and K level in raw edible portions ranged in (0.64 ± 0.02–27.0 ± 6.24), (0.46 ± 0.02–0.85 ± 0.02), (24.49 ± 1.2–131.7 ± 8.3), (0.11 ± 0.01–0.46 ± 0.04), (39.13 ± 0.34–57.27 ± 0.94), (7.34 ± 0.42–20.42 ± 1.31), and (184.4 ± 1.31–816.3 ± 11.731) mg/100 g FW, respectively. Although statistically significant losses in minerals as a result of household preparation practices were observed, the amount of nutrients retained could be valuable especially in communities that have limited alternative sources of these micronutrients. The predicted minerals' bioavailability shows adequacy in terms of calcium and zinc but not iron.

## 1. Introduction

Numerous wild edible plant species have been used by different communities in Ethiopia, mainly as supplement to conventional foods [1–8]. However, the biodiversity is threatened through replacement of forests with agricultural expansion and deforestation without cultivation and domestication of potential species [2]. These situations could exacerbate local food shortages and aggravate widespread malnutrition in the country. Diversification of production and consumption habits to include a broader range of plant species, particularly those currently identified as underutilized, could significantly contribute to improve health and nutrition, livelihoods, and ecological sustainability. Edible wild and traditional vegetables have played an important role in supplementing staple foods by supplying trace elements, vitamins, and minerals [9]. As wild food plants grow in natural conditions, they are easily accessed and freely harvested for their human food and nutrition values. They are relevant in household food security and nutrition in some rural areas, particularly during seasonal food shortage periods, and provide good nutritional supplies, notably micronutrients [10].

Micronutrient deficiencies affect billions of people globally. Although less prevalent in higher-income populations, these deficiencies do occur in such groups, especially among premature infants, children, and the elderly. Several sources revealed that edible wild plant and traditional vegetable species increase the nutritional quality by providing minerals, fiber, vitamins, and essential fatty acids and enhance taste and color in rural diets [11]. Underutilized green leafy vegetables are a good source of many nutrients like iron, calcium, ascorbic acid, and *β*-carotene that could help in overcoming micronutrient malnutrition and easily accessed by the community at a low cost. Because micronutrient deficiencies (such as vitamin A) are associated with low intake of foods such as vegetables, as opposed to starchy (energy rich) staples which provide the majority of energy intake in typical African diets, increment in energy production and consumption will likely do little to ameliorate the problem of micronutrient deficiency unless identification, proper evaluation, and domestication of nutritionally potential lesser known vegetables are integrated into the diets of the population. Moreover, the roles of edible wild plants and lesser known crops in human nutrition are potentially valuable to maintain a balance between population growth and agricultural productivity, particularly in the tropical and subtropical areas of the world [12]. Hence, continuous search for new source of nutrient especially from plant foods is a basis for selecting promising species for further studies on green leafy vegetables to meet the nutritional requirements. Evaluation of the nutrient and antinutrient compositions of wild edible plants helps to identify foods rich in minerals and acquiring knowledge on the methods of appropriate preparation to enhance bioavailability of nutrients. As the consumption of plant products low in bioavailable minerals is high in rural communities of Ethiopia [13], the presence of antinutritional factors that limits the optimal utilization of wild and traditional vegetables and the extent to which the household food preparation methods could reduce them need investigations. Therefore, the objective of this work was to assess mineral contents and their bioavailability as a function of local household preparation methods of some wild and traditional vegetables of the Gumuz ethnic community located in Benishangul Gumuz state western Ethiopia.

## 2. Materials and Methods

The study plants were collected from Agalo Meti district in Kemash zone of Benishangul Gumuz Regional state located at 530 km west of the capital Addis Ababa.

### 2.1. Selection of Species

Selection of species for our investigation was based on ethnobotanical study findings in the study area [4].* Dioscorea abyssinica *(tuber),* Oxytenanthera abyssinica *Munro (young shoot),* Abelmoschus esculentus *(L.) Moench (immature pod), and* Portulaca quadrifida *(aerial part) were selected based on their wider utilization by the community in the study area. The first two species are wild and the last two exist both under cultivation and wild stand. The selected vegetables were recommended for domestication according to an association in Benishangul Gumuz called “*Tikuret le Gumuz Limat Mahiber*” for the reason that they are widely consumed by the community and because they are most suited for domestication. Each species was collected at the time when edible parts are acceptable for consumption by the local community. Voucher specimens of the selected edible plants were collected and processed for verification. The specimens were identified by a botanist from Bioversity International and Ethiopian Public Health Institute (EPHI) using standard procedures and deposited in the National Herbarium of Addis Ababa University (AAU).

### 2.2. Sample Collection and Preparation

**Figure 1:**
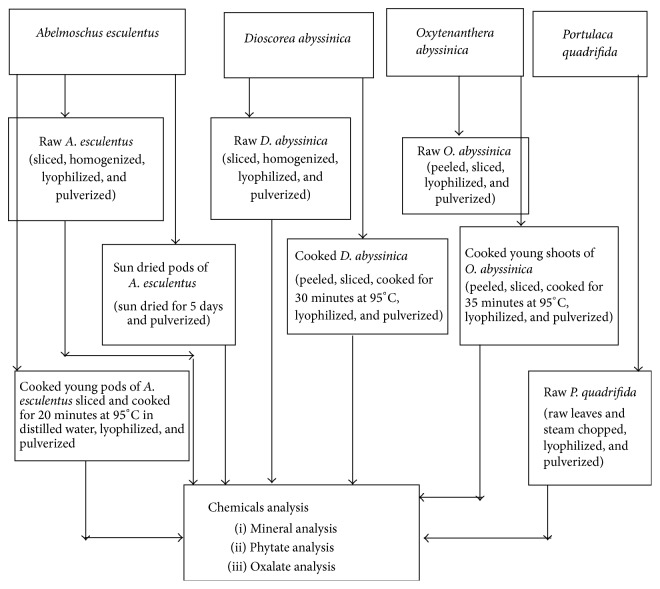
Scheme of sample preparation for chemical analysis.

Edible parts of the selected vegetables were harvested manually from their natural habitat in different areas of Agalo Meti woreda. Five-kilogram edible parts of the respective species were collected from 20 different plants of the same species to ensure the representativeness. Sample preparation for laboratory analyses is illustrated in Figure 1.

### 2.3. Mineral Analysis

The methods of [14] were used to determine minerals. The sample extract solution was transferred to polyethylene bottle and stored until use for determinations of minerals. Blank was prepared without sample by taking the same amount of reagents under the same condition. The minerals, namely, calcium, iron, zinc, and Cu, were analyzed using Shimadzu atomic absorption spectrophotometer (AA-6800/“AA Wizard” software). Sodium and potassium contents were determined using flame photometer (Jenway, PF 7, Essex, UK) according to the method described by AOAC 2005, 966.16 and 965.30, respectively. Phosphorus was determined using UV-visible spectrophotometer (CECIL Instruments, Cambridge England, deuterium F 500 mA, power T3. 15 A) based on method 970.39 [14]. Absorbance of standard, blank, and samples was read at 660 nm using UV-visible spectrophotometer. Absorbance versus concentration calibration curve was constructed and the equation obtained was used to calculate the unknown phosphorus concentration in the samples:

(1) Phosphorus in mg/100 gm=As−AB∗dilution factor∗extracted volume∗100Slope∗weight of sample∗1000,

where *A*_s_ is the absorbance of sample, *A*_B_ is the absorbance of blank, and Slope is calculated from the calibration curve.

### 2.4. Determinations of Antinutritional Factors

#### 2.4.1. Phytic Acid

The phytate content was determined as described by [15]. Briefly, 0.5 gm of dried sample was extracted with 10 mL of 0.2 N HCl for 1 hour at an ambient temperature and centrifuged (3000 rpm for 30 m). The clear supernatant was used for the phytic acid determination. Two mL of wade reagent (0.03% FeCl_3_·6H_2_O and 0.3% sulfosalicylic acid) was added to 3 mL of the supernatant sample solution. The solution mixture was homogenized and centrifuged (3000 rpm/10 minutes). The absorbance was measured at 500 nm using UV-visible spectrophotometer. The amount of phytic acid was calculated using phytic acid standard curve prepared in the same condition and the result was expressed as phytic acid mg/100 g dry weight.

#### 2.4.2. Oxalate

The oxalate content of the samples was determined using titration method [16, 17]. Two g of finely ground samples was placed in a 250 mL conical flask containing 190 mL of distilled water. Ten mL 6 M HCl solution was added to each of the samples and the suspension was digested at 100°C for 1 h. The samples were then cooled and made up to 250 mL mark of the flask. The samples were filtered and duplicate portion of 125 mL of the filtrate was measured into beaker and four drops of methyl red indicator was added, followed by the addition of concentrated NH_4_OH solution (drop wise) until the solution was changed from pink to yellow color. Each portion was then heated to 90°C, cooled, and filtered to remove the precipitate containing ferrous ion. Each of the filtrates was again heated to 90°C and 10 mL of 5% CaCl_2_ solution was added to each of the samples with stirring consistently. After cooling, the samples were left overnight. The solutions were then centrifuged at 2500 rpm for 5 min. The supernatants were decanted and the precipitates were completely dissolved in 10 mL 20% H_2_SO_4_. The total filtrates resulting from digestion of 2 g of each of the samples were made up to 200 mL. Aliquots of 125 mL of the filtrates were heated until near boiling and then titrated against 0.02 M standardized KMnO_4_ solutions to a pink color which persisted for 30 sec. The oxalate contents of each sample were calculated [18]. The analysis was carried out in duplicate, and the results were expressed in dry basis.

### 2.5. Minerals Bioavailability

Molar ratios of antinutrient/minerals were used to predict the minerals bioavailability [19]. The suggested critical values used to predict the bioavailability were calcium : phytate < 6, phytate : iron > 1, phytate : zinc > 15, and phytate : calcium/zinc > 0.5 [19, 20].

### 2.6. Statistical Analysis

Descriptive statistics such as means and standard deviation were calculated using SPSS version 16 software. One-way analysis of variance (ANOVA) was used to see the effect of household processing methods on mineral contents and the bioavailability of minerals in selected vegetables. Multiple comparison tests using least significant difference technique (LSD, *P* < 0.05) were applied to compare the means of each parameter between different household preparation practices using SPSS version 16 software. Paired comparison *t*-test was used to determine if there was a significant mean difference between raw and processed vegetables for each parameter.

## 3. Results and Discussion

### 3.1. Results

From the results presented in Table 1, it is noticeable that the concentration of different macroelements (Ca, P, Na, and K) and trace elements (Fe, Zn, and Cu) of the wild and traditional vegetables was high and the vegetables studied could be regarded as appreciably important sources of these essential elements. Potassium is abundant in all vegetables analyzed followed by calcium, phosphorus, and sodium from the macroelements. From the trace elements, iron level was relatively higher followed by zinc and copper.

### 3.2. Effect of Processing on Minerals Content of Young Pods of* Abelmoschus esculentus*

As presented in Table 2, the iron and zinc contents of raw* A. esculentus* (okra) were 2.3 mg/100 g and 0.68 mg/100 g, respectively. Iron content in immature pods of raw* A. esculentus* obtained in the present study was higher than findings of [21] for different* A. esculents* varieties reported within the range of 0.87–0.97 mg/100 g FW but the zinc content (0.68 ± 0.04 mg/100 g FW) obtained in this study was slightly lower compared to the value reported (1.29–1.37 mg/100 g FW) in their study. The variations in the contents of the minerals in* A. esculentus* may be due to the varietal difference or genetic factor, environmental factor, and their interactions [21]. Significant loss of iron and zinc was observed in the cooked* A. esculentus* but not statistically significant between raw and sun dried young pods of* A. esculentus*. This could be due to leaching of the minerals into the cooking water. Sun drying is the gradual loss of water through evaporation and cannot support leaching. It has to also be noted that minerals are not volatile.

### 3.3. Effect of Cooking on Minerals Content of* Dioscorea abyssinica*

Iron level in raw (whole)* Dioscorea abyssinica* was 27 mg/100 g FW. However, cooking and peeling the bark of the tuber significantly reduced the iron content to 1.79 mg/100 g FW. The iron content of* D. abyssinica* obtained in this study in the raw sample was higher when compared with the value reported for* Dioscorea pentaphylla* (8 mg/100 g FM) [22]. Zinc concentration of* D. abyssinica *was also significantly reduced during cooking and debarking from 0.5 mg/100 g to 0.3 mg/100 g FW. The statistically significant (*P* < 0.05) reduction of iron and zinc concentration upon cooking and peeling of the tuber may be because of the presence of minerals in the outer nonedible part of the tuber which was removed by peeling after boiling the tuber. The same reason may apply to the minerals which showed enormous reduction in the boiled and peeled* D. abyssinica*. Reduction in zinc content was also reported in peeled and boiled tubers of* Dioscorea cayenensis *[23]. The potassium level in raw tubers of* D. abyssinica* was 341.16 mg/100 g. Statistically significant losses were observed when the tubers were cooked compared to the raw samples. This loss may be attributed to the leaching out of minerals including potassium into the cooking water [24]. The high potassium content in this tuber could help to maintain normal blood pressure and can be labeled as heart protective vegetable.

### 3.4. Effect of Cooking on Minerals Content of Young Shoots of* Oxytenanthera abyssinica*

The minerals content of raw and cooked juvenile shoots of* Oxytenanthera abyssinica* is presented in Table 1. The iron and zinc contents of juvenile shoots of* O. abyssinica* were 0.6 mg/100 g and 0.9 mg/100 g, respectively. The zinc content was higher in raw bamboo shoots compared to other study vegetables. All minerals tested were decreased in the cooked samples with the reduction being statistically significant in Zn, Cu, P, Na, and K but Fe and Ca were not significantly (*P* < 0.05) lost in the cooked samples. The variation in the degree of loss might be related to the chemical forms of minerals in the food matrix. The respective potassium contents determined in raw and cooked samples of* O. abyssinica* were 456.2 mg/100 g and 273.2 mg/100 g on fresh weight basis. The potassium content of raw* O. abyssinica* obtained in the current study is well agreed with the value reported for other bamboo species such as* Bambusa tulda *(408 mg/100 g, FW) and* Dendrocalamus hamiltonii *(416 mg/100 g, FW) [25]. Raw shoots of* O. abyssinica* had the highest phosphorus content compared to other study vegetables. However, cooking has significantly reduced the phosphorus level. Phosphorus is a major component of bones and teeth [26].

Studies confirmed that there is no known benefit of high sodium consumption. Sodium intakes more than 1 g per day tend to aggravate a genetically determined susceptibility to hypertension, and intakes above 7 g/day may induce hypertension even in individuals who have no specific genetic susceptibility [27]. In this context, all the vegetables contained safe sodium levels.

### 3.5. Potential of the Study Vegetables in Meeting the RDA Requirements of Some Minerals

The recommended dietary allowance (RDA) for iron is 10 mg/day for adults [28]. The raw immature pods of* A. esculentus* could provide 116.5% of the RDA requirement for iron if 500 g fresh vegetable is consumed per day, considering it would be fully bioavailable. The cooked and sun dried* A. esculentus* could provide 34% and 129% RDA requirement for iron from 500 g FW meal. Cooked* D. abyssinica* and bamboo shoots can also provide 89.5% and 30% RDA requirement of iron from fresh 500 g meal, respectively, and only 125 g fresh leaves and steams of* Portulaca quadrifida* can provide 100% RDA requirement for iron. This RDA calculation did not consider the inhibitory effect of different antinutritional factors that affect the bioavailability of iron. The RDA for zinc is 12 mg/day for lactating woman [28]. Five hundred grams of meal (fresh basis) from cooked* A. esculentus* could provide 23% of the zinc RDA requirement for lactating mothers. Similarly, cooked* D. abyssinica* and juvenile shoots of* O. abyssinica* in the cooked form could provide 12.1 and 22.5% RDA requirement for zinc, respectively.* Portulaca quadrifida* could provide 35% of RDA for zinc considering the requirement for lactating woman. Calcium content ranged from 22.9 mg/100 g FW in cooked bamboo shoot to 140.4 mg/100 g FW in sun dried* A. esculentus*. Raw immature pods of* A. esculentus* contained 131.7 mg/100 g fresh weight basis. The calcium level obtained in this study for* A. esculentus* grown in Ethiopia was higher than the values reported for different* A. esculentus* varieties in Nigeria [21]. Raw aerial part of* P. quadrifida* contained higher (118 mg/100 g FW) calcium content next to sun dried and raw* A. esculentus*. The calcium content in raw and cooked* O. abyssinica* was 24.5 mg/100 g and 22.9 mg/100 g FW, respectively. The calcium content reported in India for the different bamboo species was within the range of 21.17–180.69 mg/100 g (29); the present value obtained for* O. abyssinica* was similar to* Bambusa balcooa* (24.01 mg/100 g). The calcium content shows no significant (*P* > 0.05) variation between raw and boiled* D. abyssinica* and* O. abyssinica* (Table 1). However, significant reduction was observed when* A. esculentus* was cooked but sun drying did not affect the calcium content. The RDA for calcium is 1000 mg/day for adults [28]. Consumption of 500 g cooked* A. esculentus*, cooked* D. abyssinica*, and* O. abyssinica *and raw* P. quadrifida* can contribute 52.12, 20.37, 11.5, and 59.0% RDA requirement for calcium, respectively.* Abelmoschus esculentus* and* P. quadrifida* are good source of calcium. Copper was the element found in a trace amount in all wild and traditional vegetables analyzed which ranged from 0.07 to 0.46 mg/100 g FW. This is important due to the fact that copper is required by body at a trace level for many metabolic activities (25). Phosphorus content in this study ranged from 23.7 mg/100 g to 57.3 mg/100 g FW. Raw* A. esculentus, D. prehensilis*, and* O. abyssinica* contained 39.1, 40.7, and 57.3 mg/100 g phosphorus, respectively. Cooking resulted in significant losses (7.3–41.76%) of phosphorus with variable degree of proportion among the vegetables (Table 1). Similar findings were reported in the earlier studies [23]. RDA for phosphorus is 700 mg/day for adults [28]. Consumption of 500 g cooked* A. esculentus*,* D. abyssinica,* and* O. abyssinica *and raw* P. quadrifida* could contribute by meeting 25.9, 16.9, 27.9, and 28.4% RDA requirement for phosphorus, respectively.

### 3.6. Bioavailability of Minerals

Antinutritional components such as oxalates, tannins, polyphenols, and phytic acid (myoinositol hexaphosphate) present in plant foods are known to have adverse effects on human nutrition by inhibiting iron [29] and zinc [30] absorption. The molar ratios along with the suggested critical values for predicting the bioavailability calcium, iron, and zinc are presented in Table 2.

### 3.7. Molar Ratio of Phytate to Zinc

The calculated phytate/zinc molar ratios for raw and processed WEPs were within the range of 2.03–14.22, which were in the range of the suggested critical level (<15 regarded as favorable for zinc absorption) [19]. Ratios ≥15 are associated with low zinc bioavailability [19]. According to WHO cut-offs phytic acid to zinc mole ratio ≥15, 5–15, and <5 is equal to zinc bioavailability as low (10–15%), moderate (30–35%), and high (50–55%), respectively [31]. In this context,* A. esculentus* and* P. quadrifida* had moderate zinc bioavailability, whereas the molar ratios suggested that high zinc bioavailability could be achieved from* D. abyssinica* and juvenile shoots of* O. abyssinica*. However, the critical phytate : zinc molar ratio may also depend on dietary calcium levels because of the kinetic synergism between calcium and zinc ions resulting in Ca : Zn : Phy complex which is less soluble than phytate complexes formed by either of the ions alone [32], suggesting that Ca : phy/Zn molar ratio is better predictor of zinc bioavailability than Phy : Zn molar ratio alone.

### 3.8. Molar Ratio of Calcium × Phytate/Zinc

Calcium × phytate/zinc molar ratios of cooked and sun dried* A. esculents *and* P. quadrifida *were above the critical level (0.5 mol/Kg) as indicated in Table 2, suggesting that calcium interference was more likely to affect zinc bioavailability. The Ca × Phy/Zn molar ratio reported in the earlier study for different* A. esculentus* varieties in Nigeria was within the range of 0.293–0.436 [21]. Higher calcium content in* A. esculentus* that grows in Ethiopia compared to Nigeria could be attributed to genetic differences, environmental variability, and interaction of the genetic factor with the environment. Considering both Phy/Zn and Ca × Phy/Zn molar ratios, zinc could adequately be absorbed in the body from* D. abyssinica* and shoots of* O. abyssinica*.

### 3.9. Molar Ratio of Phytate to Iron

As indicated in Table 2, phytate/iron molar ratios were >1 (indicative of poor iron bioavailability) for all raw and processed study plants except* D. abyssinica.* This might be due to the reported higher phytate content and insufficient phytic acid degradation (4.5–27.5%) by boiling alone. Phytate reduction (13–33%) after boiling* Dioscorea *sp. was reported by [23].* Dioscorea abyssinica* could be a better source of bioavailable iron.

### 3.10. Molar Ratio of Calcium to Phytate

The calcium/phytate molar ratios in all the raw and processed study plants were >6, which is regarded as favorable for calcium absorption [33], predicting that a good calcium bioavailability could be achieved from all the selected vegetables. In addition to phytic acid, oxalic acid in insoluble form is responsible for interference of divalent metals absorption particularly calcium by forming insoluble salts [34]. The oxalate/calcium molar ratios of all the selected raw and processed vegetables were below the critical level of 2.5 known to significantly impair calcium bioavailability suggesting that they are good calcium bioresources for the local populace.

## 4. Conclusions

Immature pods of* Abelmoschus esculentus*, aerial parts of* Portulaca quadrifida,* and juvenile shoots of* Oxytenanthera abyssinica *consumed by the Gumuz community indicated that they are appreciably important sources of essential minerals. Except* D. abyssinica*, the wild and traditional plant parts have significant micronutrient compositions. However, cooking significantly reduced some of the minerals. Reducing duration of cooking time and using other processing methods such as fermentation (*D. abyssinica* and* O. abyssinica*) might alleviate the deterioration of the nutrients. The predicted mineral bioavailability shows adequacy in terms of calcium and zinc (moderately bioavailable) but not in iron. Hence, there is a need for some enhancers to increase iron absorption in all species. The study results further revealed that* A. esculentus *and* P. quadrifida *are rich sources of bioavailable calcium. With the exception of* D. abyssinica*, the vegetables are rich in potassium and can contribute in maintaining normal blood pressure and its heart protective role.

To be able to justify the overall nutritional value of the wild and semiwild edible vegetables, proper assessment of the type and concentration of their antinutrients is necessary. The rate in reduction of antinutritional factors depended upon the type of processing (cooking and sun drying) and vegetable. However, the reduction of phytic acid, oxalate, and tannins by traditional cooking methods alone was not adequate to the level that could improve iron and zinc bioavailability. The vegetables have higher moisture content and are mostly used after cooking as side dishes by the community. The combination effects of these factors might reduce the actual impact of antinutritional factors in impairing bioavailability of nutrients and/or causing ill health. However, appropriate processing methods that are known to reduce the antinutritional factors (such as fermentation) could be encouraged in the community.

## Figures and Tables

**Table 1:** Mineral content (mg/100 g, fresh basis) of raw and processed vegetables.

Species		Fe	Zn	Ca	Cu	P	Na	K
*A. esculentus*	RAS	2.33 ± 0.37^b^	0.68 ± .04^b^	131.7 ± 8.3^b^	0.11 ± 0.01^b^	39.14 ± 0.95^b^	7.82 ± 0.28^b^	184.4 ± 1.3^b^
	CAS	0.68 ± 0.08^a^	0.55 ± 0.03^a^	104.23 ± 4.9^a^	0.08 ± 0.01^a^	36.28 ± .067^a^	4.4 ± 0.29^a^	155.4 ± 1.7^a^
	SAS	2.58 ± 0.16^b^	0.54 ± 0.02^a^	140.4 ± 8.13^b^	0.08 ± 0.01^a^	51.85 ± 0.34^c^	7.51 ± 0.92^b^	183.3 ± 53.4^b^

*D. abyssinica*	RDP	27.0 ± 6.24^b^	0.46 ± 0.02^b^	43.19 ± 2.0^a^	0.46 ± 0.04^b^	40.68 ± 2.7^b^	8.94 ± 0.54^b^	341.16 ± 3.6^b^
	CDP	1.79 ± 0.06^a^	0.29 ± 0.06^a^	40.74 ± 6.8^a^	0.19 ± 0.02^a^	23.69 ± 2.4^a^	5.88 ± 0.75^a^	128.49 ± 3.9^a^

*P. quadrifida*	RPO	8.06 ± 0.11	0.84 ± 0.06	117.99 ± 10.8	0.14 ± 0.01	39.13 ± 0.34	20.42 ± 1.31	816.3 ± 11.7

*O. abyssinica*	ROA	0.64 ± 0.02^a^	0.85 ± 0.02^b^	24.49 ± 1.2^a^	0.11 ± 0.01^b^	57.27 ± 0.94^b^	7.34 ± 0.42^b^	456.2 ± 12.3^b^
	COA	0.6 ± 0.06^a^	0.54 ± 0.02^a^	22.94 ± 4.21^a^	0.07 ± 0.01^a^	39.7 ± 1.89^a^	4.20 ± 0.55^a^	273.2 ± 1.6^a^

RAS = raw, CAS = cooked, and SAS = sun dried; RDP = raw, CDP = cooked, RPO = raw, ROA = raw, and COA = cooked; values are expressed as mean ± SD of three determinations; mean values in the same column corresponding to each species followed by different superscript letters were considered significant at *P* < 0.05.

**Table 2:** Antinutrient/mineral molar ratios of raw and processed vegetables.

Species		[Phy]/[Zn]^1^	[Ca]/[Phy]^2^	[Phy]/[Fe]^3^	[Phy × Ca]/[Zn]^4^	[Ox]/[Ca]^5^
*A. esculentus *	RAS	6.077 ± 0.45^a^	51.11 ± 0.77^a^	1.50 ± 0.001^a^	1.73 ± 0.12^a^	0.17 ± 0.001^b^
	CAS	5.50 ± 0.23^a^	57.20 ± 0.35^b^	4.07 ± 0.35^b^	1.57 ± 0.06^a^	0.15 ± 0.001^a^
	SAS	7.48 ± 0.03^b^	53.04 ± 0.77^a^	1.68 ± 0.06^a^	2.11 ± 0.1^b^	0.15 ± 0.001^a^

*D. abyssinica*	RDP	3.79 ± 0.19^a^	35.0 ± 0.50^a^	0.06 ± 0.001^a^	0.10 ± 0.01^a^	1.04 ± 0.001^b^
	CDP	3.66 ± 0.60^a^	45.63 ± 0.84^a^	0.51 ± 0.01^b^	0.09 ± 0.01^a^	0.28 ± 0.001^a^

*P. quadrifida*	RPO	14.22 ± 0.39	15.06 ± 0.12	1.28 ± 0.02	3.21 ± 0.07	0.72 ± 0.002

*O. abyssinica*	ROA	2.03 ± 0.10^a^	22.64 ± 0.51^a^	2.22 ± 0.11^a^	0.12 ± 0.01^a^	0.75 ± 0.002^b^
	COA	3.74 ± 0.10^b^	24.55 ± 0.09^a^	1.81 ± 0.13^a^	0.22 ± 0.0^b^	0.50 ± 0.01^a^

Critical values		*15.0*	*6.0*	*1.0*	*0.5*	*2.5*

RAS = raw, CAS = cooked, and SAS = sun dried; RDP = raw, CDP = cooked, RPO = raw, ROA = raw, and COA = cooked. Values in the same column followed by the same superscript corresponding to the same species are not significantly different (*P* < 0.05).

^1^(mg phytate/MW of phytate : mg Zn/MW of Zn), ^2^(mg Ca/MW of Ca : mg phytate/ MW of phytate),^ 3^(mg phytate/MW of phytate : mg Fe/MW of Fe), ^4^(mol/Kg of phytate × mol/Kg of Ca : mol/Kg of Zn), and ^5^(mg oxalate/MW of oxalate : mg Ca/MW of Ca).
